# Right ventricular myocardial infarction and pulmonary embolism differential diagnosis –a challenge for the clinician

**Published:** 2010-08-25

**Authors:** C Ginghina, GA Caloianu, M Serban, D Dragomir

**Affiliations:** * >Emergency Institute for cardiovascular diseases ‘C.C.Iliescu’, BucharestRomania; ** Emergency Hospital of MAI ‘Prof Dr. D. Gerota’, BucharestRomania

## Abstract

It is frequently recognized in medical literature as well as in daily clinical practice that right ventricular myocardial infarction and pulmonary embolism are two of the most challenging clinical pictures to differentiate in cardiology and the treatment, often chosen upon a mixture of clinical suspicion criteria subsequently confirmed by other diagnostic methods, can lead to therapeutic success. Differential diagnosis is often difficult due to similar clinical picture, unspecific electrocardiographic changes and unspecific biological markers. It is very important to know the risk factors and the associated comorbidities for these two clinical entities in order to be able to interpret them contextually. In most cases the diagnosis key is the clinical suspicion. Usually in evaluating these cases we are in the position of choosing more complex diagnostic procedures, most likely not available in Emergency Department. In conclusion it is expected from the clinician to use the available methods with a thorough approach to details but in the same time considering the whole clinical picture.

It is frequently recognized in medical literature as well as in daily clinical practice that the right ventricular myocardial infarction (RVMI) and pulmonary embolism (PE) are two of the most challenging clinical pictures to differentiate in cardiology and the treatment, often chosen upon a mixture of clinical suspicion criteria subsequently confirmed by other diagnostic methods. They can lead to therapeutic success.

Though for some time is has been thought to be less important, RVMI, is actually considered a condition that can lead to a worsening of the whole cardiac function. It was noticed that in 10 to 50% of patients, a RVMI was associated with inferior myocardial infarction, the large difference in percentage being explained by the different diagnosis criteria used. Isolated RVMI was found in no more than 2 to 5% of the autopsies. [1,3]

Due to the structural and functional characteristics of the right ventricle (thin walled chamber with low pressure, low oxygen demand and both systolic and diastolic perfusion), it is unusual to have a massive extension of the infarction and an irreversible progress. More frequently transient systolic dysfunction and reversible myocardial stunning are seen and most of the cases are recovering the right ventricular function in time. [1,3,5]

In evaluating these cases, in order to differentiate between RVMI and PE we are in the position of choosing more complex diagnostic procedures, most likely not available in the Emergency Department. 

## Risk factors and associated comorbities

It is very important to know the risk factors and the associated comorbidities for these two clinical entities in order to be able to interpret them contextually. The risk factors for myocardial infarction are well known (hypertension, dyslipidemia, smoking, diabetes). Half of the patients with RVMI seem to have identified a precipitant factor (such as intense physical activity, psychological stress, and post surgical massive blood loss) as well as prodromal symptoms. [1,3,5]

## Clinical picture

In most cases, the diagnosis key is the clinical suspicion. The clinical picture of RVMI often mimics the PE presentation. In clinical practice, this explains the high proportion of diagnosis errors (aprox 70–75% of the cases).

Clinically, RVMI is frequently associated with inferior or posterior myocardial infarction and presents with:

- angina–like pain or epigastric pain
- clinical triad – hypertension, raised jugular venous pressure, lack of pulmonary stasis – is relatively specific and it is considered characteristic for RVMI by some authors,  but sensitivity is less than 25% as it is also frequently present in PE.
- marked sensitivity preload reducing agents (nitrates, morphine, diuretics)
- brady–arrhythmias and high degree AV block
- right ventricle wall rupture and cardiac tamponade are quite rare

An increased awareness must be kept in mind when we are using these elements because, very often they can be masked by volume depletion and the specific signs are usually obvious only after correcting the fluid status. [1,3,5, 6–11]

The specific signs identified in patients with PE are not as specific and they are useful to confirm PE only by associating them with the clinical context (Table 1). [2]

**Table 1:** Clinical symptoms and signs described in pulmonary embolism [2]

Symptoms	Approximate prevalence
Dyspnoea	80%
Thoracic pain (pleuritic)	52%
Thoracic pain (substernal)	12%
Cough	20%
Syncope	19%
Haemoptysis	11%
Physical signs	
Tachypnoea (>20/min)	70%
Tachycardia (>100/min)	26%
Signs of deep vein thrombosis	15%
Cyanosis	11%
Fever (>38.5)	7%

In a classical and systematically approach, patients with RVMI have signs of right heart failure and those with PE have signs of pulmonary hypertension and only in some cases signs of right heart failure.

In patients with clinically suspected PE, the initial clinical evaluation is concomitant with the risk stratification and clinical probability assessment. That can be estimated by using a validated score (Geneva, Wells – Table 2 and Table 3) or by global clinical evaluation. More frequently, a franc clinical picture of deep vein thrombosis with lower limb oedema makes the diagnosis of PE more probable (15% of the cases) [2, 12–16]

**Table 2:** Revised Geneva Score[2]

Variables	Score
Predisposing factors	
age >65	+1
previous deep vein thrombosis or pulmonary embolism	+3
surgery/fracture within 1 month	+2
active malignancy	+2
Symptoms unilateral lower limb pain at palpation or unilateral oedema	+3
haemoptysis	+2
Clinical signs heart rate 75–94 bpm	+3
>95bpm	+5
lower limb tender deep vein or unilateral oedema	+4
Clinical probability	Total
low	0–3
moderate	4–10
high	>11

**Table 3:** Wells Score [2]

Variables	Score
Predisposing factors	
history of deep vein thrombosis /pulmonary embolism	+1,5
recent surgical intervention or immobilization	+1,5
neoplasm	+1
Symptoms haemoptysis	+1
Clinical signs	
heart rate >100bp	+1,5
clinical signs of deep vein thrombosis	+3
Clinical diagnosis alternative diagnosis less likely than pulmonary embolism	+3
Clinical probability	Total
low	0–1
moderate	2–6
high	>7
Clinical probability	
unlikely	0–4
likely	>4

## Electrocardiography

The electrocardiography (ECG) can bring many important diagnostic elements that can help us differentiate between RVMI and PE but only in 30 to 40% of cases, these changes are specific. In almost one third of the patients with both conditions, the electrocardiogram is within normal limits. [4,5]

It is important to remember that in all cases of suspected RVMI right chest leads recording has to be precociously obtained because, very often the ST changes are transient and they are not being recognizable after 24– 48 hours after the onset of the symptoms. [1,3,5,6–11]

**Figure 1:**
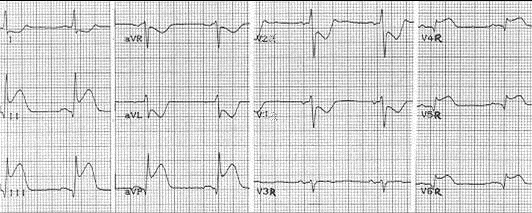
Electrocardiography in a case of inferoposterior and right ventricular myocardial infarction: 65bpm sinus rhythm, QRS axis at 60 degrees, elevation of ST segment in  DⅡ, DⅢ, aVF, V4R–V6R  and ST depression V1–V2

The ST elevation with more than 0,5mm in V4R lead is highly sensitive for RVMI. However, this has low specificity when an anterior left ventricular myocardial infarction is not excluded. Anyway, in RVMI, there is usually a progressive ST elevation from V4R to V3 that can be useful in differentiating from an anterior myocardial infarction. (Figure 1) [17, 20]

Andersen and co. have analysed the role that V3R – V7R recording are playing in identifying the RVMI concluding that  the ST elevation with 1mm or more in V3R has a 81% specificity and a 77% positive predictive value. When that was associated with a ST segment elevation in another lead, such as V4R–V7R, the specificity and positive predictive value increased to 100%. [27]

Moreover, the presence of a Q wave in V3R has a positive predictive value of 83% in identifying RVMI. The same study shows that the ST elevation with 1mm or more in right chest leads has only a 28% sensitivity in anterior MI. However, we must not forget that the right chest leads ST changes in RVMI can be masked when a massive left ventricle one is associated. [18, 27]

One study by Lewin and co. looked exclusively at Q wave changes finding that small Q waves in DⅡ, DⅢ and aVF  associated with tall R waves in the same leads with a R/Q ratio more than 2.5 are highly sensitive (80%) and specific (70%) for RVMI. In cases with biventricular infarction, Q waves become deep and R waves are small, R/Q ratio being less than 2.5. [18, 19]

**Figure 2:**
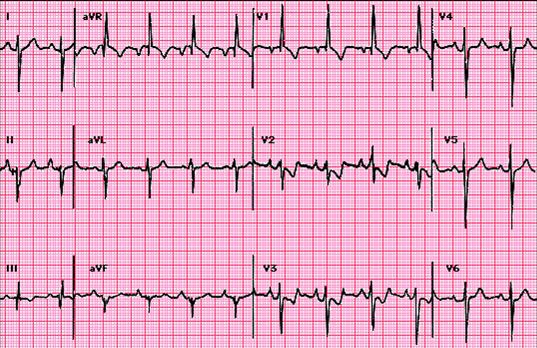
Electrocardiography in a case of pulmonary embolism: 95bpm sinus rhythm, QRS axis at –150grd, major right bundle branch block, typical S1, Q3, T3 aspect.

In PE ECG, changes are frequently non–specific and transient and they are less prominent when haemodynamic status is corrected. One study published by Ferrari and co. showed that the presence of the T wave inversion in V1–V4 is the most frequent change, being present in 68% of the cases of PE. [22] The other features of PE are the signs of right heart overloading such as those presented in (Table 4). Sometimes arrhythmias are also present (extra systoles, atrial fibrillation). (Figure 2) [21–24]

**Table 4:** ECG diagnosis criteria for pulmonary embolism (modified from Chou) [21]

Typical changes
S1Q3 or S1Q3T3
Right QRS axis deviation
Right bundle branch block or transient incomplete right bundle branch block
T wave inversion in chest leads
Other changes
left transition – clockwise rotation
QR in V1
R>5mm in V1 or R/S>1 in V1
ST elevation in DⅠ or DⅡ
ST elevation in DⅢ
ST changes or T wave changes in left chest leads
ST changes in right chest leads
right atrial overload – p ‘pulmonale’
sinus tachycardia or atrial arrhythmias (atrial tachycardia, extra systoles, flutters)
1st degree atrioventricular block
limb leads small complexes

As a rule, when a characteristic electrocardiogram is present, it is suggestive of PE, but a normal aspect does not exclude the diagnostic.

## Biological profile

Cardiac markers: The usual blood tests may not bring any useful elements to differential diagnosis between RVMI and PE. However, the cardiac markers for necrosis can help, generally by using CK, CK–MB and troponins. They reflect the lesion but not its mechanism, and, the raised levels can indicate a myocardial infarction only if the clinical picture is suggestive for the diagnostic. Though CK MB is usually not present in the lungs, it was showed that it could be raised in 7.7% of the cases. This represents a marker of right ventricular necrosis secondary to right heart pressure overload, due to PE (40–55% of the cases). [1, 3, 28, 29]CK MB with a value twice as normal has specific dynamic changes in order to be significant in diagnosing RVMI. In the first few hours from the onset, this has a sensitivity of only 25–50% that increases to 60–100 % after 4 hours, with a specificity of 85–100%. [1,3,5]Cardiac troponins T and I are highly sensitive and specific markers in identifying myocardial lesion, and, they are routinely used in diagnosing acute coronary syndromes. The single values are determined according to the context, so, they have a 39% sensitivity and 93% specificity but these can be increased to 90–100% for specificity and 83–96% for sensitivity when serial determinations of troponin I are used. [1,3,5] Donketis and al. showed that raised troponin 1 levels are present in 20,8% of the patients with massive PE (quoted values between 7 and 42%), but they have a low sensitivity and a low diagnostic value. These changes do not exclude PE in patients with thoracic pain or dyspnoea. [2, 30, 31]Increased troponin levels have also a prognostic value both in RVMI and PE and raised levels indicate a higher risk for complications, higher mortality and longer hospital admission. [3, 32,33]D–Dimers: One of the most important tests in diagnosing PE is D–dimer level with a high sensitivity of 96%. Though they are fibrin specific, raised levels are present in necrotic lesions, neoplasm, inflammatory processes and pregnancy. [2]. A normal D–dimer value has a low predictive value for PE and suggests a low probability. These values must be judged in the diagnosis algorithm and the associated clinical probability assessment should be revised by applying valid scores such as Wells or Geneva.  [3, 4, 34]Other biological markers: The other laboratory tests such as slightly raised ESR; moderate leucocytosis;  raised plasma fibrinogen; raised hematocrit; global hypercoagulative status in the first 48 h from the onset are not specific neither for RVMI nor for PE. In patients with PE one can also encounter normal AST; raised total LDH and raised iso–enzymes (3,4,5); raised total bilirubin and especially indirect bilirubin – in the first 2–3 days from the onset. Additional laboratory tests can be requested in young patients with RVMI or PE in order to establish their coagulation status. [2–4].In clinical practice, there are markers that are frequently associated with the mortality and the degree of right ventricle dysfunction such as uric acid; natriuretic peptide – pro BNP and NT fraction; norepinephrine; endoteline.  [3, 35–37]Blood gases: Non–specific changes are usually present and they should not be used for diagnosis. In RVMI, the arterial blood gases can be normal but in massive infarction secondary hypoxemia, they can be present due to low cardiac output. The hypoxemia can be corrected by oxygen–therapy. When oxygen therapy does not correct the hypoxemia in a RVMI, an intra–cardiac right to left shunt should be suspected (patent foramen ovale usually being present). [3,7,8] In PE, mild to moderate arterial hypoxaemia is often present as a consequence of a reduced cardiac output or an impaired ventilation–perfusion ratio. When severe hypoxaemia is associated, a massive PE or a right–to–left shunt is present. (i.e. patent foramen ovale – same as in RVMI). A normal oxygen pressure does not exclude a PE. Low CO2 values can often be present due to hyperventilation associated with a mild degree of respiratory alkalosis. An alveolo–arterial ratio higher than 15–20 is a more sensitive indicator for PE when compared to the oxygen pressure.  [2,4,12,13]

## Chest X–ray

The routine chest X–ray has a limited value in the differential diagnosis of the two conditions and the changes are ranging from a normal aspect to specific changes.

In RVMI, the cardiac shadow is increased secondary to cardiac enlargement, pulmonary stasis is absent and superior vena cava and /or inferior vena cava dilatation may be present. Generally, radiographic changes are present in late stages and they are not helpful in the acute management needed in both situations. When the right myocardial infarction is associated with the left ventricle one, a degree of pulmonary stasis may be present due to global systolic impairment. [3,5, 7–9]

Chest X–ray can bring helpful information in PE when it shows features of pulmonary hypertension. These depend on the dimensions, the site of the embolus and the time past from the onset (Table 5). Both the sensitivity and the specificity of the chest X–ray are low. [2,4 12–13]

**Table 5:** Chest X–ray findings in PE (2,4, 12–14)

Normal X–ray in 10–20% of cases
X–ray changes suggestive of PE (proximal site without pulmonary infarction; associated high risk ) with following features: unilateral hyperinflation increased pulmonary hilar shadows due to pulmonary artery dilatation Westmark sign ( increased vascular markers in the opposite lung) cardiac and vessels changes typical signs of lobar/segmental artery obstruction in 5–10% of the cases elevated diaphragm on the affected side	X–ray changes where pulmonary infarction is associated (distal site) periferic shadow more often in the shape of a triangle small pleural effusion on the same side elevated diaphragm on the same side

**Figure 3:**
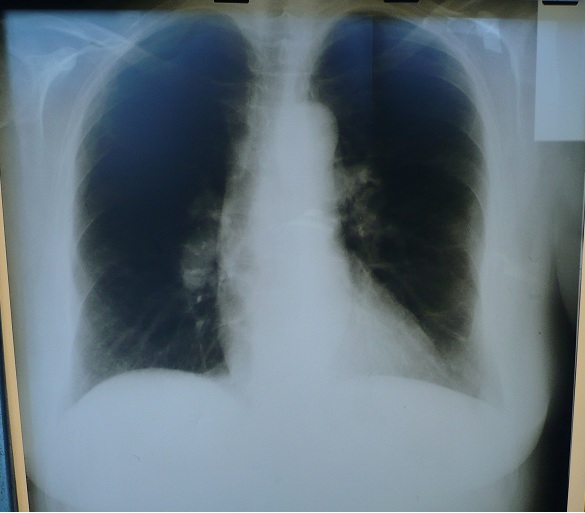
Postero anterior cardiopulmonary radiography in a case with pulmonary embolism: cardiothoracic index within normal limits, bilaterally enlarged pulmonary hilum with amputated aspect and peripheral oligohemia

The right cardiac enlargement with a raised cardio–thoracic index and a round cardiac shape can also be found. If a lateral view is a performed, a cardiac shadow moves anteriorly occupying the retro–sternal area.

## Echocardiography

Because of the challenges that the differential diagnosis poses, based on clinical assessment, performing an echocardiogram is very important. In RVMI, a short parasternal view and an apical incidence that can evaluate all four cardiac chambers and wall motion are of great use. In RVMI, the right cardiac chambers are dilated and they present motion abnormalities. They are usually associated with inferior and posterior left ventricle wall dyskinesia. Defects in the motion of the interventricular septum can be present during systole (paradoxical movement) as well as in diastole (suggesting high right ventricle pressures with inverted trans–septal pressure gradient). [3, 8, 17, 40–41]

**Figure 4:**
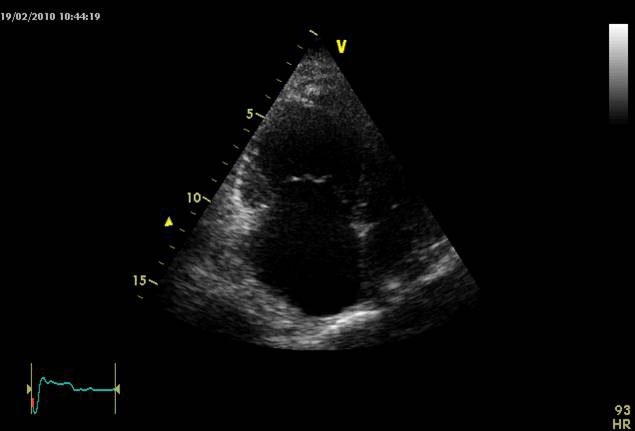
2D echocardiogram  four chamber apical section: right cavities dilated, leftward shift of the interventricular septum (pulmonary embolism).

**Figure 5:**
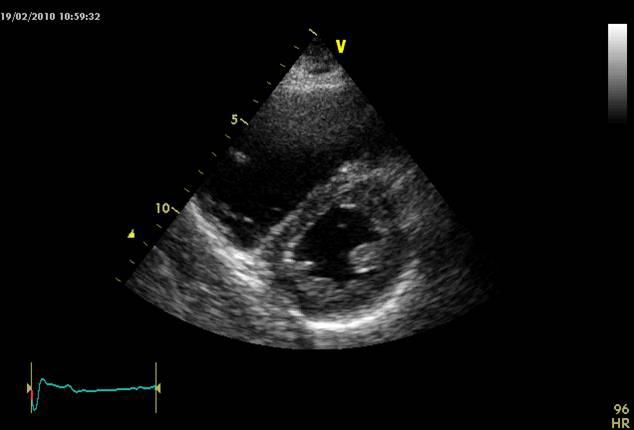
2D echocardiography parasternal short axis view at the level of papillary muscles: important right ventricular dilatation, leftward shift of the interventricular septum (pulmonary embolism).

A right ventricle infarction can range from small hypo–kinetic areas to marked, extensive dilatation and systolic dysfunction. Sometimes, a leftward displacement of the interatrial septum can be present in massive right ventricle infarction or if an atrial infarction is associated, suggesting right heart increased pressures. Inferior vena cava dilatation can be present and respiratory variations are usually lacking (Figure 4, Figure 5) [8, 40–45]

**Doppler examination** is useful in RVMI, showing, in most of the cases, the presence of a tricuspid valve dysfunction and dilatation. In extensive RVMI, ventricular filling and ejection flows are very slow and they last for almost the entire cardiac cycle. This pattern, similar to that of a vein, indicates that right ventricle pumping function is lost and it is now behaving as a conduit with a passive role. [8, 40–42]

The complexity of the right ventricular structure makes the evaluation of the ejection fraction difficult. The systolic and diastolic function of the right ventricle can be evaluated by using the right ventricle myocardial performance index, with a value >0.3 suggesting the presence of a RVMI. An 82% sensitivity and a 95% specificity are associated with this finding. A reliable marker for the right ventricular function is TAPSE (tricuspid annular plane systolic excursion) as it evaluates the longitudinal cardiac function. This method is particularly useful in RVMI cases. [8,41–43]

Some of these findings are not characteristic for right myocardial ventricular infarction and can be found in PE as well.

Frequently, right cardiac chambers are dilated in PE, the interventricular septum is flattened or even a leftward displacement is seen creating a D shape left ventricular aspect. A paradoxical movement of the interventricular septum can be found secondary to the right ventricle overloading. Inferior vena cava dilatation without respiratory variations is also present. [3,12–13, 39]

A distinct pattern of right ventricular systolic dysfunction with right ventricular free wall severe dyskinesia and with normal apical contractility is described in PE (McConnel sign). A 77% sensitivity and 94% specificity is associated with its presence in PE and the positive predictive value is 77%, while, the negative predictive value is 96%. Casazza and co., however, showed that this sign is not useful in the differential diagnosis of these two conditions. [38]

**Figure 6:**
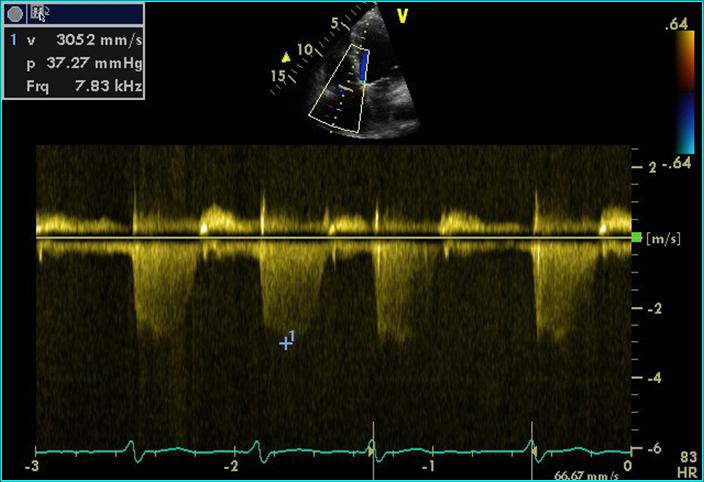
Continuous Doppler echocardiography at the tricuspid valve: Doppler recording indicating tricuspid regurgitation, with an estimated RV-RA gradient of 37mmHg (pulmonary embolism)

A relatively specific sign for PE is the increase of systolic pulmonary artery pressure (SPAP) with its value determined at tricuspid flow, a finding that is not present in RVMI. [39] (Figure 6)

**Figure 7:**
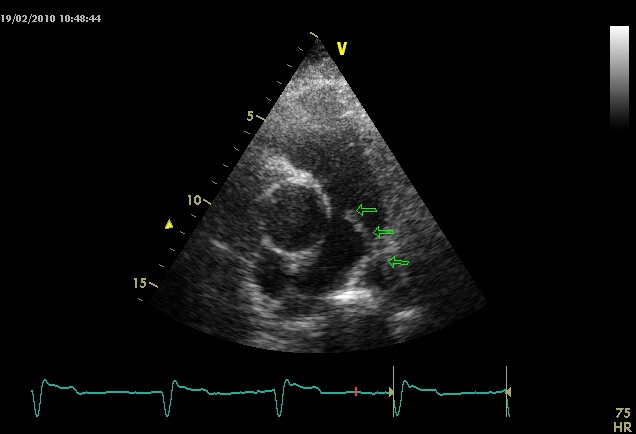
2D echocardiography parasternal short axis view at the level of great vessels: thrombus visible in pulmonary artery truncus and right branch (arrows)

Rarely, intracavitary thrombi can be found in both situations, parietal thrombi in akinetic areas are present in RVMI, while transient thrombi at the level of inferior vena cava, tricuspid valve or atrial septal defect are seen in PE. (Figure 7)

**Tissue Doppler** imaging is useful in determining myocardial velocities and for ventricular function evaluation. In RVMI, the systolic and early diastolic velocities, myocardial velocities for the free right wall are decreased. Tissue Doppler echocardiography can also demonstrate ventricular asynchrony by using septal and left ventricular free wall measurements.  [8,42–43]

In RVMI, the typical apex–base gradient is inverted and high flow velocities are found at the apex and in the outflow tract. In PE, an increase in myocardial strain–rate is noticed with the decrease of maximal systolic strain indicating a reduction in cardiac output. The profile changes from protomezosystolic to telesystolic and even protodiastolic (postsystolic shortening) [42–43]

**Figure 8:**
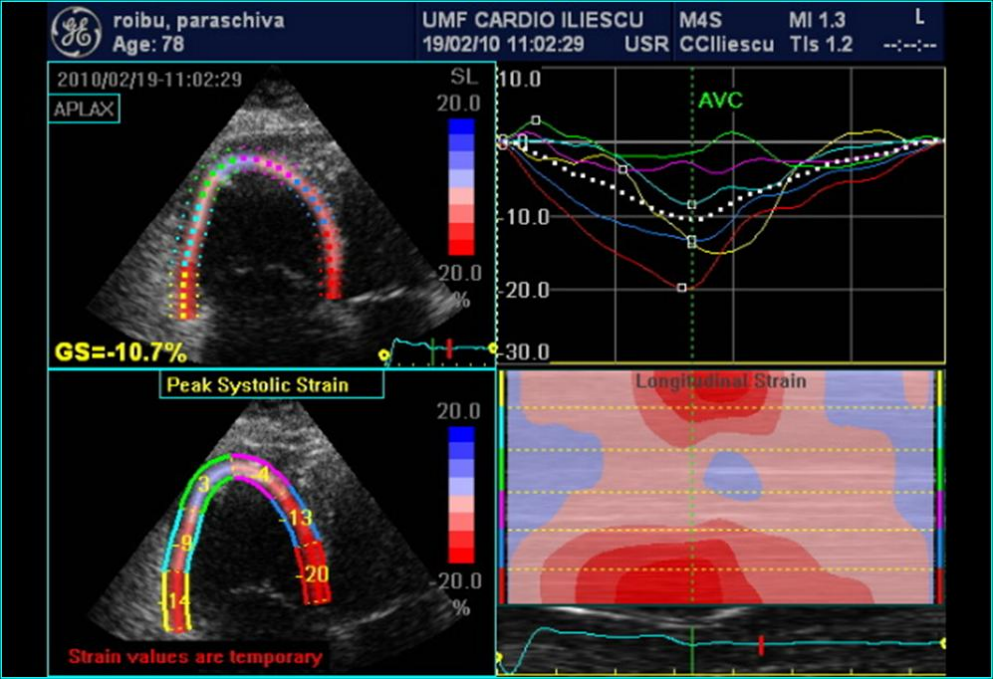
Tissue Doppler echocardiography to measure the myocardial strain in a case of  pulmonary embolism: decreased myocardial strain at the level of the right ventricle, with postsistolic shortening aspect in strain curve recorded in bazal segment of the right ventricle free wall,  interventricular septum with normal myocardial strain aspect.

**Figure 9:**
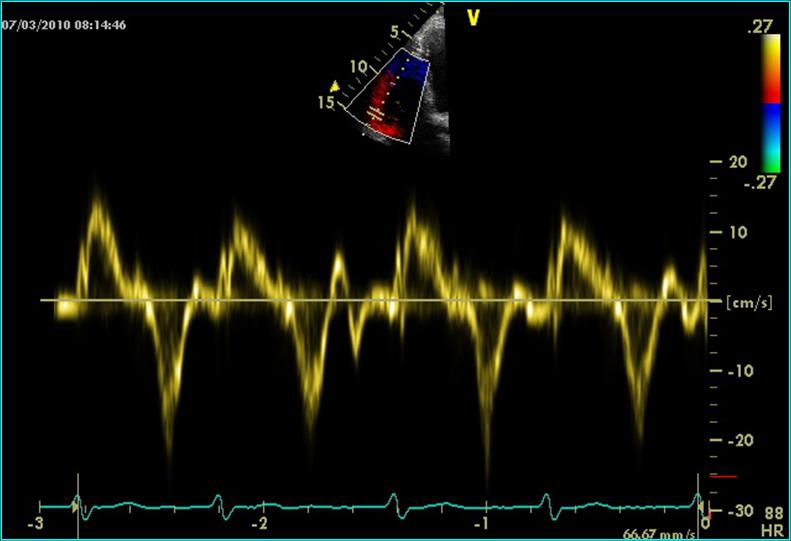
Tissue Doppler echocardiography in a case of pulmonary embolism: decreased myocardial velocity at the level of right ventricular free wall

Other sonographic imaging techniques (transesophageal echocardiography, intravascular ultrasound) are used in special situations where the clinical suspicion for a PE is high and when the diagnosis cannot be confirmed by other techniques. [8]

## Nuclear imaging

**Radionuclide ventriculography** is useful in investigating myocardial infarction as it allows the evaluation of ventricular volumes, segmental wall motion and ejection fraction. In RVMI, there is an evident significant dilatation with motion abnormalities at the level of right ventricle free wall, especially in the apical area, with a reduced ejection fraction. [3,5,8–10]

Myocardial scintigraphy is rarely used at the onset of a myocardial infarction due to difficulties in mobilisation of the patient. Technetium 99 piro–phosphate scintigraphy is useful for the diagnosis of ischemia or acute myocardial infarction and in risk stratification. The necrotic area is evidentiated as a result of myocardial concentration of the radiolabeled drug at this level. However, this method has a low sensitivity especially in those cases with inferior localisation of the infarction. [5, 7–11]

Scintigraphic imaging of myocardial perfusion (using Thallium 101 or Technetium 99m sestamibi) is a sensitive technique in diagnosing myocardial infarction. This technique, however, cannot differentiate between acute necrosis and old myocardial infarction and, can also have false negative results in those cases with small necrosis. (myocytic loss of<10g) [5, 8]

**Pulmonary perfusion scintigraphy** is useful in diagnosing PE. The data can be classified into three categories:

1. Normal (excluding PE)
2. Positive – high probability – affecting more than one segment (PE confirmed)
3. Non–diagnostic with equivocal result when PE cannot be excluded nor confirmed

There are correlations between the degree of clinical suspicion and evaluation of the scintigram results. When the diagnostic is more difficult, additional methods can be used. **Ventilation scintigraphy** can show normal ventilation associated with perfusion defects evidentiated by perfusion scintigraphy and a significant impairment ventilation/perfusion ratio is directly correlated with the degree of vascular obstruction. [2, 4, 12–14]

## Computer tomography (CT)

**Figure 10:**
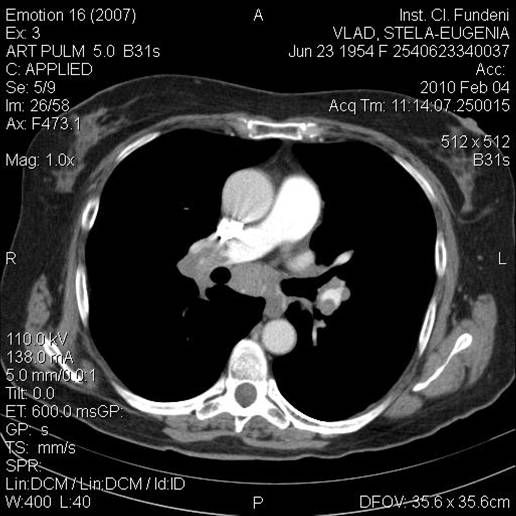
Chest CT scan using contrast substance is showing a filling gap at the level of left branch of pulmonary artery consistent with pulmonary thrombembolism

In PE, CT scan with or without contrast (spiral angio CT) is very useful for diagnosis, as it can show the intraluminal thrombi in main pulmonary arteries or in segmental and subsegmental branches with a sensitivity of 75–95%. In some cases beside the intra–arterial filling defects, CT scan can show the presence of a pulmonary infarction, usually a triangular shadow with its tip towards the hilum and the base towards the pleura. In other cases, alveolar haemorrhages can be present with a consolidation–like appearance or an oedema –like aspect. [2,4, 12–14, 44](Figure 10)

In the meanwhile, the CT scan is not so helpful in evaluating patients with myocardial infarction. Right ventricle dilatation may be present in those cases. [45]

## Magnetic resonance imaging

Magnetic resonance imaging it is used to evaluate cardiac function and actually represents the ‘golden standard’ investigation in the determination of ventricular volumes and wall motion. Magnetic resonance imaging can rapidly identify (within 1 hour) those changes present in an acute myocardial infarction, and, it is the only method that can really differentiate between subendocardial and the transmural infarction. [46–50]

**Figure 11:**
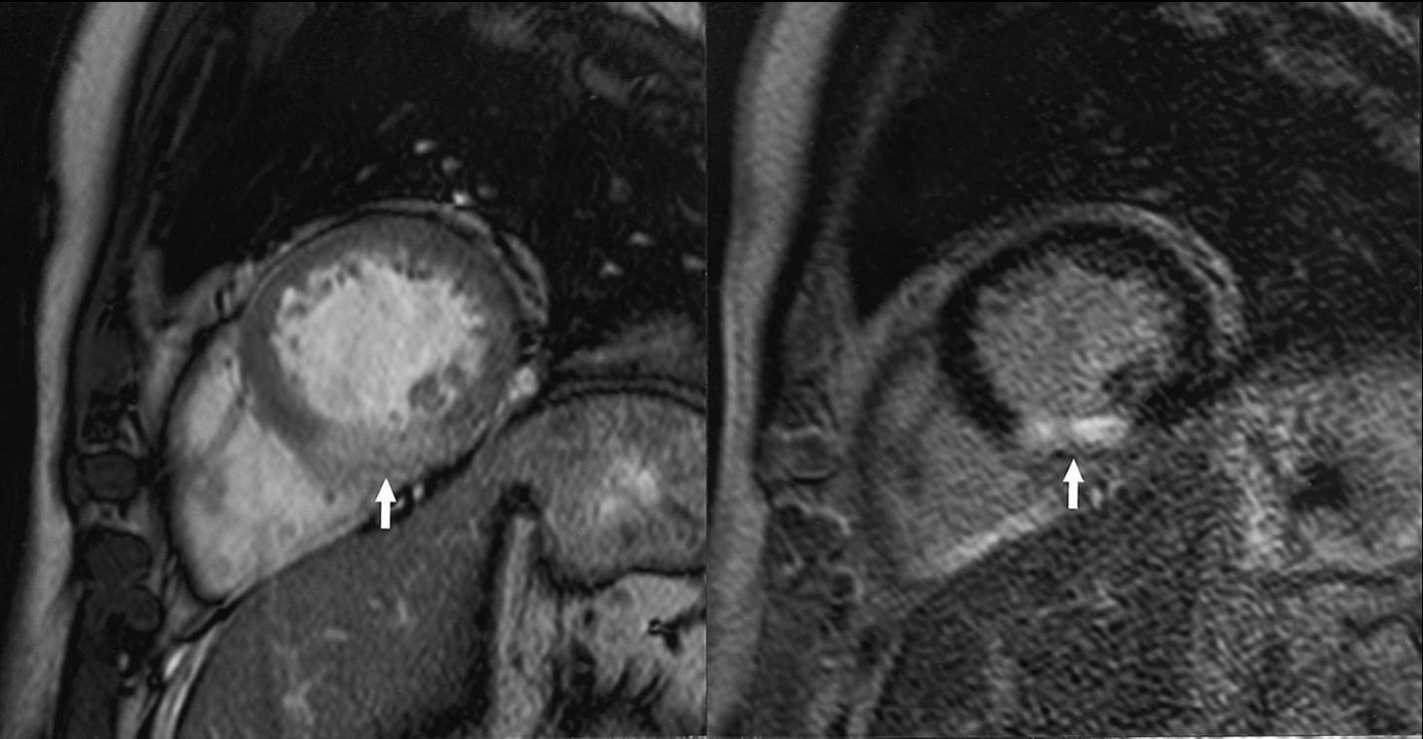
Magnetic resonance imaging in a case of acute myocardial infarction - early and tardive acquired images show increased signal in the inferoseptal area with transmural distribution.

In PE, both the sensitivity and specificity of magnetic resonance imaging are lower when this method is compared with angio–CT scan to detect the pulmonary artery thrombi. Magnetic resonance imaging can evaluate both pulmonary artery flows as well as cardiac flows, but the method is not routinely used remaining at this moment an alternative to CT scan. [3, 46–50]

## Invasive investigations

**Coronary arteriography** is not routinely used in diagnosing, but it is more frequently used to identify the coronary lesions more precisely, with the precise scope of performing primary angioplasty. [1,3,5]

**Pulmonary arteriography** has a higher accuracy in identifying pulmonary artery obstruction, being for the moment the diagnostic ‘gold standard’ method. Because it is an invasive method, it is not routinely used; being performed only in those cases where the diagnostic is not confirmed and all the other methods cannot bring sufficient information, or where a therapeutic approach such as an endovascular intervention is considered. [2, 4]

In RVMI, these changes are not present unless it is complicated in evolution with intracavitary thrombosis and secondary pulmonary embolisation.

## Other diagnostic methods used in the investigation of clinically suspected PE

**Doppler venous ultrasound, Contrast venography; Impedance pletismography** are useful in evaluating patients with clinically suspected PE, as it can show thrombi in the lower limbs venous system or in other territories.

**Figure 12:**
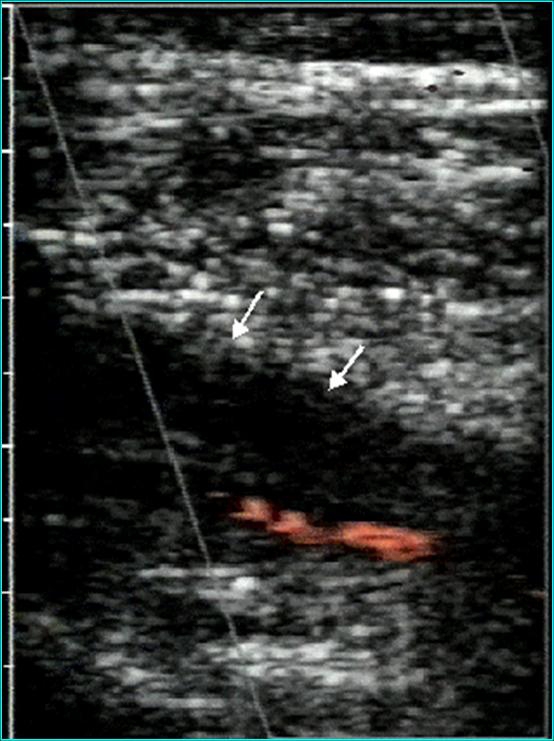
Venous Color Doppler examination: hyper echogen material present in superficial femoral vein which is partially permeable (aspect suggestive for old nonoclusive thrombosis)

## Conclusions

Differentiating between RVMI and PE can be very difficult in clinical practice. It is important to perform a through investigation and all the information has to be looked at in detail and ultimately integrated in the final complex picture of the case. 

In conclusion, the clinician is expected to use the available methods wisely in order to make a differential diagnosis between the right ventricle myocardial infarction and PE, with a thorough approach to details, but in the same time, considering the whole clinical picture. 
